# The Impact of Handedness, Sex, and Cognitive Abilities on Left–Right Discrimination: A Behavioral Study

**DOI:** 10.3389/fpsyg.2018.00405

**Published:** 2018-03-27

**Authors:** Martin Constant, Emmanuel Mellet

**Affiliations:** ^1^Institut des Maladies Neurodégénératives (IMN), UMR 5293, University of Bordeaux, Bordeaux, France; ^2^Centre National de la Recherche Scientifique (CNRS), Institut des Maladies Neurodégénératives (IMN), UMR 5293, University of Bordeaux, Bordeaux, France; ^3^CEA, Groupe d’Imagerie Neurofonctionnelle, Institut des Maladies Neurodégénératives (IMN), UMR 5293, University of Bordeaux, Bordeaux, France; ^4^Institut des Maladies Neurodégénératives (IMN), UMR 5293, Team 5: GIN Groupe d’Imagerie Neurofonctionnelle, Centre Broca Nouvelle-Aquitaine, Bordeaux, France

**Keywords:** left–right discrimination, handedness, sex difference, cognitive abilities, visuo-spatial, language

## Abstract

The present study examined the relationship between left–right discrimination (LRD) performance and handedness, sex and cognitive abilities. In total, 31 men and 35 women – with a balanced ratio of left-and right-handers – completed the Bergen Left–Right Discrimination Test. We found an advantage of left-handers in both identifying left hands and in verifying “left” propositions. A sex effect was also found, as women had an overall higher error rate than men, and increasing difficulty impacted their reaction time more than it did for men. Moreover, sex interacted with handedness and manual preference strength. A negative correlation of LRD reaction time with visuo-spatial and verbal long-term memory was found independently of sex, providing new insights into the relationship between cognitive skills and performance on LRD.

## Introduction

The ability to discriminate left from right, called left–right discrimination (LRD), is essential in everyday life. Whether the task is following directions to an unknown place or operating on a patient’s knee, it is necessary to be able to differentiate left from right. It is reasonable to assume that such an essential ability would be mastered by most people. However, many people report difficulties discriminating left from right in daily life (Hannay et al., 1990), resulting in what we call Left–Right Confusion. Moreover, very few healthy people have trouble discriminating up from down (Hirnstein et al., 2009). The lack of difficulty in up–down discrimination may be due to the strong up–down asymmetry of our world, induced by gravity (Vingerhoets and Sarrechia, 2009). Hence, this spatial confusion phenomenon seems specific to left–right discrimination. LRD can be divided into two types: egocentric and allocentric (Auer et al., 2008). Egocentric LRD is the ability to discriminate left from right from one’s own perspective with typical orientations. Allocentric LRD is used for unusual orientations or for other people’s bodies and is said to be an association of egocentric LRD with mental rotation. The present work used the Bergen Left–Right Discrimination Test (BLRDT, Ofte and Hugdahl, 2002a,b), which focuses on allocentric LRD.

Left–right discrimination can be assessed through different measures. Older studies (e.g., Hannay et al., 1990) often used self-report questionnaires exclusively (questionnaires on subjective LRD performance in daily life). Most recent studies have used behavioral tasks such as BLRDT instead of self-report, or coupled self-report questionnaires with behavioral tasks (e.g., Jordan et al., 2006). Those studies identified several factors explaining LRD variability, including sex, handedness and education.

Sex differences in self-reported left–right confusion are found repeatedly. According to the results of questionnaires, women are more prone to left–right confusion compared with men (Hannay et al., 1990; Jordan et al., 2006; Gormley et al., 2008; Hirnstein et al., 2009; Hirnstein, 2011; Ocklenburg et al., 2011; Slagman, 2014; McKinley et al., 2015). However, performance reported by women is inconsistently correlated to their performance of actual behavioral tasks, with some studies reporting moderate correlation between the two (Gormley et al., 2008; Vingerhoets and Sarrechia, 2009; Thomas et al., 2013; Grewe et al., 2014; McKinley et al., 2015) and others reporting no correlation (Jordan et al., 2006; Hirnstein et al., 2009). Compliance with sex stereotypes may be the reason for women’s lower scores on self-reports (Jordan et al., 2006). Some studies also find sex differences when behavioral tasks are analyzed (Ofte, 2002; Ofte and Hugdahl, 2002a; Gormley et al., 2008; Hirnstein et al., 2009; Ocklenburg et al., 2011; Hjelmervik et al., 2015; McKinley et al., 2015) but other studies do not find such differences (Hirnstein, 2011; Hirnstein et al., 2011; Thomas et al., 2013; Grewe et al., 2014). Finally, one study reported sex differences in behavioral tasks only in participants from 18 to 22 years old. In studies with a wider age range, a sex difference has either not been found or not been reported in older adults (Ofte and Hugdahl, 2002a). Thus, the effect of sex on left–right discrimination remains an open issue, with one possible explanation being that sex interacts with other factors such as handedness. The sample of participants in the present study was balanced for sex and handedness, thus maximizing the possibility that evidence for such an interaction could be found.

Another factor affecting LRD may be handedness. Indeed, Hannay et al. (1990) found that right-handers reported fewer difficulties than left-handers in LRD. Moreover, Ofte (2002) found that left-handed men performed better than right-handed men on the BLRDT. Yet, there are many studies that do not report a significant difference between left-handers and right-handers (Jaspers-Fayer and Peters, 2005; Jordan et al., 2006; Gormley et al., 2008; Vingerhoets and Sarrechia, 2009; Grewe et al., 2014; Slagman, 2014; McKinley et al., 2015). It should be noted, however, that there are no studies with a ratio of left-handers above 15%. Moreover, most studies are based solely on self-reports, which have proven to be unreliable (Jordan et al., 2006). In addition, Vingerhoets and Sarrechia (2009) demonstrated that handedness had no impact on its own but that stronger manual preference strength and asymmetry were correlated with better performance. We aimed to investigate more thoroughly the debated difference in LRD between left-handers and right-handers by including a high ratio of left-handers (41%) and taking into account the strength of handedness. We also investigated sex difference and its potential interaction with handedness. Additionally, Marzoli et al. (2015) demonstrated that in a task with pictures of ambiguous human silhouettes performing one-handed manual action, both left- and right-handers were more prone to say that the silhouettes were performing the action with the right hand. The authors hypothesized that both left- and right-handers had “an attentional bias toward the right-arm.” Therefore, unlike previous LRD studies, we aimed to determine whether such a bias could be found in LRD.

Finally, the cognitive abilities factor was explored. Ofte and Hugdahl (2002a) found that children younger than 8 years old exhibit the lowest LRD performance (12%). Adolescents (12–13 years old) and older adults (*M* = 67) had better performance (40%). Young adults (18–22) had significantly better performance than all groups (60%). This finding supports the assumption that LRD is a developmental skill (Piaget, 1929; Elkind, 1961). Moreover, LRD performance seems to follow the same declining trend as spatial cognitive abilities in older adults (Techentin et al., 2014). Benton (1968) proposed that one component of LRD is visuo-spatial ability. Accordingly, students’ academic curriculum has been found to influence their left–right discrimination performance, with medical students performing better than law and psychology students (Ofte, 2002). The fact that medical students are more proficient at LRD and have stronger spatial abilities strengthens the potential relation between LRD and spatial cognitive performance. In addition, medical students who wanted to be surgeons had better LRD scores than those wanting to be general practitioners or medical doctors (Gormley et al., 2008). It was hypothesized that this enhancement may be due to the more frequent use of spatial abilities among future surgeons than among other medical students. Note, however, that the nature of the visuo-spatial skills related to strong performance in LRD remains to be defined, as previous studies failed to demonstrate a relationship between LRD and scores on a Mental Rotations Test or between LRD and a navigation task in a 3D virtual maze (Jordan et al., 2006; Ocklenburg et al., 2011). In addition, Benton suggested that LRD entailed a verbal component, including the attribution of words to the concept of left and right. However, the relationship between verbal ability and LRD performance has been poorly investigated in adults.

Consequently, we investigated the relation of visuo-spatial cognitive abilities and LRD using more tests and extended this assessment to verbal cognitive abilities, the underlying hypothesis being that the participants with more developed abilities would either have shorter reaction times or make fewer errors.

Unlike most studies, which use behavioral tasks to assess proficiency in LRD, we focused not only on the error rate but also on the reaction time. Previous studies using the BLRDT used a pen-and-paper version, with limited time to complete a maximum number of items. We used a computerized version with one stimulus at a time and no total time limit. Our assumption was that LRD performance could be measured by both accuracy and processing time.

In summary, this work intended to unravel the relationships between sex, handedness and abilities in language, verbal memory and visuo-spatial domains in left–right discrimination. So far, the effect of these factors has been studied apart. It has previously been shown that interactions between these factors could affect cognitive performances (Mellet et al., 2014a). The present work investigated whether such interactions could also affect performances in left–right discrimination, which could explain the lack of consensus regarding their role in the inter-individual variability of LRD. In addition, the parameters of the task which modulated its difficulty such as the number of arms crossings or the orientation were included in the analysis.

## Materials and Methods

### Participants

The study was approved by the Basse-Normandie local Ethics Committee CPP Nord-Ouest III. 66 participants (31 men, 35 women) were included in this study and underwent the BLRDT. Extensive cognitive testing was available in 55 participants who belonged to the BIL&GIN database (Mazoyer et al., 2016). The mean age was 24.5 ± 4.5 years for women and 25.5 ± 7 years for men. The mean level of education (years since first grade) was 16.1 ± 2 years for women and 15.8 ± 2.2 years for men.

### Procedure and Tests

#### Handedness

The participants were asked to self-report their handedness. According to the responses, there were 27 left-handers (41%, 14 women, 13 men) and 39 right-handers (59%, 21 women, 18 men), which is well above the typical population ratio for left-handers (∼10%; Annett, 1970; Hécaen, 1984).

Handedness was further assessed by the Edinburgh Handedness Inventory (Oldfield, 1971). The test comprises 10 items assessing the preferred hand of the participant in daily use and in the manipulation of various objects and tools. The BIL&GIN version of the Edinburgh Handedness Inventory was used with the “broom” item excluded since very few young people had enough familiarity with this tool. A score of -100 indicates strong left-handedness whereas a score of +100 indicates strong right-handedness.

The left-handers’ score ranged from -100 to +17.6, with a mean of -58.6 ± 39.8. For right-handers, the range was +25 to +100 with a mean of +90.3 ± 18.1. The fully lateralized (-100 or +100) represented 64.1% (25 participants) of the right-handers and 33.3% (9 participants) of the left-handers. Such a difference is consistent with other studies (Mellet et al., 2014a); a common explanation is the fact that left-handers are under-represented in the general population (∼10%; Annett, 1970; Hécaen, 1984) and have to adapt to a right-handers’ world. Finally, 18 women were not fully lateralized and 17 were fully lateralized.

#### Assessment of Cognitive Skills

Fifty-five among 66 participants performed a series of 10 cognitive tests.

Four tests assessed their spatial cognition. The first test was the Mental Rotation Test (Vandenberg and Kuse, 1978), which assesses performance in mental rotation. The second was the Raven Progressive Matrices (Raven, 1956) test, which assesses non-verbal reasoning. The third was the Corsi block-tapping test (Della Sala et al., 1999), which assesses visuo-spatial working memory.

The fourth test was an in-house virtual maze test that assesses topographic memory. The participants first had to memorize a survey perspective 2D map of the maze, which contained seven items. They then switched to a 3D route perspective of the maze, and the examiner asked them to retrieve items in a specific order. When they retrieved an item, they were given the name of the next item to retrieve. The score is dependent on the number of items retrieved and the time spent to retrieve each item.

Two tests assessed verbal long-term memory. First, the participants performed a custom version of Rey’s 15 words list (Rey, 1958). The custom version had 18 words in order to palliate a ceiling effect observed during the non-delayed recalls. The participants listened to the list five times. At the end of each listening session, they had to recall as many words as they could. Twenty minutes later (with no verbal tasks in between), they had to recall as many words as possible from the list. The collected variable was the number of words retrieved after the 20-min lapse.

Secondly, the participants performed the same task with a list of 15 pseudo-words. The variable considered was the number of pseudo-words retrieved after the 20-min lapse.

Verbal working memory was assessed through two tasks. The first was the Reading Span Test (Daneman and Carpenter, 1980; Desmette et al., 1995), and the second was the Listening Span Test (Daneman and Carpenter, 1980).

In the Reading Span Test, the participants read sentences on a computer screen. The number of sentences increased after each block (first 3 × 2 sentences, then 3 × 3 and up to 3 × 6). The participants had to read each sentence out loud and, at the end of a block, they had to recall the last word of each sentence.

The Listening Span Test followed the same pattern, except that each sentence was read by the examiner and, instead of reading it out loud, the participant had to determine whether it was in the present tense.

The participants completed a vocabulary test (Binois and Pichot, 1956) where they had to find, in a list of 6 words, the synonym of a given word. There were a total of 46 given words.

The participants also performed a verb generation task. They heard a pre-recorded list of words, with 10 s between each word. During this lapse of time, they had to list as many verbs as they could, related to the word they heard.

#### Bergen Left–Right Discrimination Test

**FIGURE 1:**
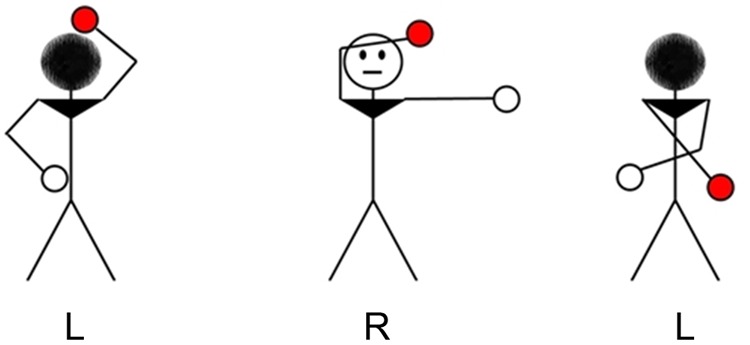
The Bergen Left–Right Discrimination Test. Participants had to decide by button press whether the labels ‘R’ or ‘L’ below the figure matched the left or right hand highlighted in red. In this example, the first item is incorrect while the second and third items are correct.

A computerized version of the pen-and-paper concrete version (Ofte and Hugdahl, 2002b) of the BLRDT (**Figure 1**) was used. Stimuli were presented electronically on a laptop using the E-Prime 2.0 software (Psychology Software Tools, Pittsburgh, PA, United States).

The stimulus set was composed of 96 line drawings of a figure; 50% of the figures were presented from the back and 50% from the front. When the head of the figure was black, it meant it was viewed from the back. When a face was drawn, it meant the figure was viewed from the front. The arms of the figure had different positions, with no, one or both arms crossing the central line of the body. The presentations of each crossing condition were balanced. The target hand (colored in red) was the left-hand half of the time, and it was the right-hand the other half of the time. Under the figures were the letters “D” or “G” (French abbreviation for Right or Left), and the participants had to determine whether the letter was congruent with the target hand. Due to a labeling error, the congruent situation was presented 47 times, and the incongruent was presented 49 times.

To answer, the participants had to press either 8 (congruent) or 5 (incongruent) on a numpad. The keys were labeled with a green sticker marked “Vrai” (True) on 8 and a red sticker marked “Faux” (False) on 5. The keys were arranged vertically to prevent a stimulus-response effect.

Stimuli were presented in a randomized order; the participants had no time restrictions and were simply instructed to answer as soon as they felt they had the correct answer. After each response, a blank screen was presented for 1000 ms before the next stimulus appeared.

The analyses entailed computing reaction times for correct answers and for error rates.

### Statistical Analysis

Statistical analysis was conducted with the JMP software (SAS, Cary, United States, version 13.2).

A total of 96 measures per participant were collected. The outliers in reaction time were excluded from the analyses using Tukey’s method, excluding values below the 1st quartile – 1.5 × Inter Quartile Range and above the 3rd quartile + 1.5 × IQR. In total, 388 (6.1%) values above 5348 ms were excluded, and 32 were wrong answers. To perform reaction time analyses, we excluded the remaining 194 wrong answers, resulting in a total of 582 (9.2%) values being excluded.

A repeated-measures 3 (Crossings) × 2 (Orientation) × 2 (Target hand) × 2 (Sex) × 2 (Handedness) × 2 (Manual Preference Strength) ANOVA on reaction times was performed. The within-participants factors were Crossings (0, 1, or 2 arms crossing the midline of the stickman), Orientation (Front or Back view) and Target hand (Right or Left). The between-participants factors were Sex, Handedness and Manual Preference Strength (MPS). MPS was set to MPS+ for participants with either -100 or +100 Edinburgh scores, and it was set to MPS- for the others.

The effect of between-subject factors on error rate was assessed with a 2 (Handedness) × 2 (MPS) × 2 (Sex) ANOVA. An outlier participant was excluded.

A 2 (Label) × 2 (Target hand) × 2 (Handedness) × 2 (Sex) repeated-measures ANOVA on reaction time was performed to determine whether the label or congruency (corresponding Label and Target hand) had an effect and whether such an effect would be influenced by Handedness or Sex.

A Principal Components Analysis (Promax rotation) was performed to reduce the resulting matrix of standardized scores from the ten verbal and visuo-spatial tests. The scree criterion was used to determine the number of factors to include. This resulted in a four-component solution that explained 60.9% of the variance.

The first was a Spatial Cognition component that aggregated the Raven matrices, the Mental Rotation Test, the maze test and the Corsi block test and explained 20.1% of the variance (loading factors: 0.92, 0.63, 0.57, 0.43, respectively). The second was a Verbal Long-term Memory component that aggregated Rey’s 18 words test and the pseudo-words test and explained 14.3% of the variance (loading factors: 0.88, 0.75, respectively). The third was a Verbal Working Memory component that aggregated the Reading Span Test and the Listening Span Test, explaining 13.3% of the variance (loading factors: 0.98, 0.42, respectively). The last was a Lexical component that aggregated the vocabulary test and the verb generation test and explained 13.3% of the variance (loading factors: 0.98, 0.32, respectively).

A multiple linear regression was computed to assess the relationship between the reaction time and the four components evidenced by the PCA. The same analysis was also conducted on the mean error rate. In the original sample of the BIL&GIN (436 adults), no differences were found between left- and right-handers on any of the cognitive components, and a significant difference was found – in favor of men – on the spatial cognition component (Mellet et al., 2014a). In our subsample, there was a significant difference in favor of men in the spatial cognition (*p* = 0.0010) and in the verbal working memory (*p* = 0.0393) components. There was a significant difference in favor of women in the verbal long-term memory (*p* = 0.0032) component, while Handedness had no significant impact on any of the components. Therefore, Sex was included as covariate in the linear regression.

## Results

### Effects of Sex, Handedness, and Manual Preference Strength

**FIGURE 2:**
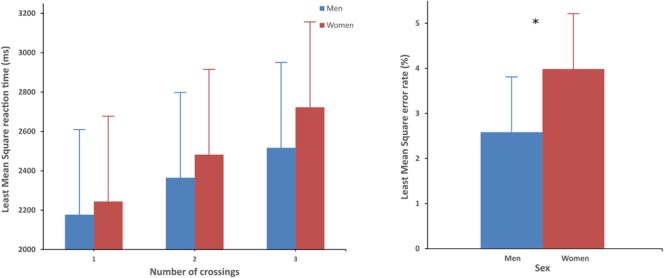
**Left**: Interaction between Sex and the number of Crossings on reaction time. **Right**: Error rates for women and men. Error bars represent inter-group Confidence Interval (95%). ^∗^Represents significant difference (*p* < 0.05).

A significant main effect of the number of Crossings was found, *F*(2,111) = 106.04, *p* < 0.0001, $\eta_{p}^{2}$ = 0.66. Tukey’s *HSD* showed that all levels were significantly different (all *p*s < 0.0001) with no crossing being the easiest, one crossing being intermediate and two crossings being the hardest. There was a significant interaction between number of Crossings and Sex, [*F*(2,111) = 3.09, *p* = 0.0493, $\eta_{p}^{2}$ = 0.05, **Figure 2**, left]. Although, no *post hoc* tests survived to the Tukeys’s correction, this interaction indicated that women’s reaction times tended to be more affected by the increasing number of crossings (i.e., the task difficulty) compared with the reaction times of men.

The ANOVA revealed a significant main effect of Orientation, *F*(1,57) = 4.94, *p* = 0.0301, $\eta_{p}^{2}$ = 0.08. Participants were faster to respond on back-view stimuli (*M* = 2374 ± 76 ms) than front-view stimuli (*M* = 2462 ± 76 ms).

No main effect of Sex (*p* = 0.3755) or Handedness (*p* = 0.1155) was found on reaction time, but interactions with intra- or between-subjects factors were evidenced (see below). A main effect of sex was evidenced on error rate: *F*(1,57) = 5.24, *p* = 0.03, $\eta_{p}^{2}$ = 0.08 (**Figure 2**, right). Women had a higher error rate (*M* = 4 ± 0.4%) than men (*M* = 2.6 ± 0.4%).

**FIGURE 3:**
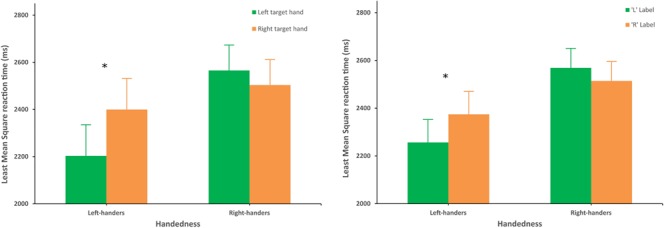
**Left**: Interaction between Handedness and Target hand on reaction time. Left-handers were significantly faster when the left hand rather than the right hand was the target. ^∗^Represents significant difference (*p* < 0.05). **Right**: Interaction between Handedness and Label on reaction time. Left-handers were significantly faster when the label “L” was presented. Error bars represent intra-group Confidence Interval (95%). ^∗^Represents significant difference (*p* < 0.05).

The interaction for Handedness and Target hand was significant on reaction time, *F*(1,54) = 16.00, *p* = 0.0002, $\eta_{p}^{2}$ = 0.23 (**Figure 3**, left). Tukey’s *HSD* showed that the left-handers were significantly (*p* = 0.0012) faster when the left hand (*M* = 2203 ± 116 ms) rather than the right hand (*M* = 2399 ± 116 ms) was the target. Correspondingly, the error rate was lower for left hands than for right hands in left-handers (2.5% and 4.6%, respectively, *p* = 0.02 paired *t*-test). No difference was found for right-handers (*p* = 0.45).

A marginally significant interaction for Sex and MPS was found, *F*(1,58) = 3.71, *p* = 0.06, $\eta_{p}^{2}$ = 0.06. Women MPS- tended to be slower than men MPS-. The interaction was more complex concerning the error rate, involving Sex, MPS and Handedness [*F*(1,57) = 7.75, *p* = 0.007, $\eta_{p}^{2}$ = 0.12]. Women MPS- made more errors than men MPS-, but this result was found among right-handers only [*Post hoc t*-test: *t*(11), *p* = 0.02].

A significant interaction between Label and Handedness (**Figure 3**, right) was revealed, *F*(1,60) = 13.09, *p* = 0.0006, $\eta_{p}^{2}$ = 0.18. A *post hoc* Tukey’s *HSD* showed that left-handers were significantly faster (*p* = 0.0104) when the label “L” (*M* = 2256 ± 109 ms) was presented rather than the label “R” (*M* = 2374 ± 109 ms). Such an advantage was also found in the error rates, which were lower for the label “L” than for the label “R” in left-handers (2.5 and 4.6%, respectively, *p* = 0.03, Wilcoxon). No difference could be found for right-handers (*p* = 0.29).

A significant interaction between Label and Target hand was also found, *F*(1,60) = 116.43, *p* < 0.0001, $\eta_{p}^{2}$ = 0.66. *Post hoc* Tukey’s *HSD* showed that participants were significantly faster (∼267 ms) when the label was congruent with the target hand (*p* < 0.0001). No such difference was found for error rate (*p* = 0.34, Wilcoxon).

### Correlation of LRD Performance With Cognitive Abilities

**FIGURE 4:**
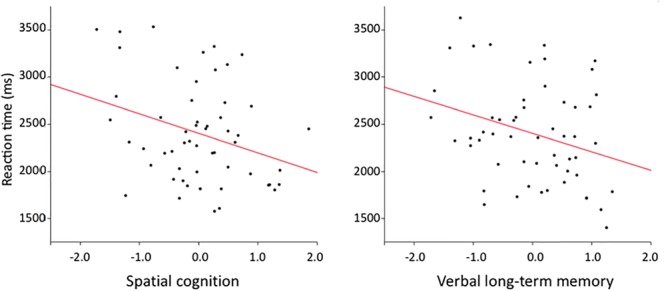
Negative correlation between the reaction times and the normalized score on the spatial cognition component **(left)** and the verbal long-term memory component **(right)**.

A significant correlation was found between reaction times on the BLRDT and cognitive abilities, *F*(5,49) = 3.4282, *p* = 0.0098, *R*^2^ = 0.26. *Post hoc* analysis revealed that reaction times to the BLRDT were negatively related to the Spatial cognition score, *t*(49) = -2.27, *p* = 0.0278, and the Verbal Long-term Memory score, *t*(49) = -2.20, *p* = 0.0326 (**Figure 4**). Participants’ mean reaction time was 2404 ms and decreased by 207 ms for each point in the spatial cognition component and by 196 ms for each point in the Verbal Long-term Memory component.

No significant correlation was found between cognitive abilities and the error rate, *F*(5,48) = 0.71, *p* = 0.40, *R*^2^ = 0.07.

### Summary of the Results

The reaction time increased significantly with the number of crossings. It also increased significantly for the front-view over the back-view.

Women had longer reaction times than men as the number of crossings increased, and they made significantly more errors than men. Not fully lateralized right-handed women made significantly more errors than not fully lateralized right-handed men.

Left-handers were significantly faster at identifying left target-hands over right target-hands, and they were significantly faster when the label “L” was presented over the label “R”. They also made significantly fewer errors on the left target-hands over the right target-hands. Both left- and right-handers exhibited a congruency effect: reaction time was significantly shorter when the label matched the target hand.

Finally, reaction times were negatively correlated with the Spatial Cognition and the Verbal Long-term Memory components of the PCA.

## Discussion

The main aim of the present study was to investigate the variability in behavioral performance differences in LRD using the Bergen Left–Right Discrimination Test (BLRDT). Unlike most of the previous studies, we focused not only on the error rate but also on the reaction time as indicators of performance. We aimed to determine whether handedness was a significant factor of variability in LRD performance by analyzing the performance of a sample with a more balanced ratio (41%) of left-handers than that used in other studies (<15%). We also investigated sex differences and the relationships between cognitive abilities and LRD.

We identified several task-related differences. The first was a significant impact of Orientation on the reaction time, even though we used the concrete version stimuli, which tend to reduce back/front differences (Ofte and Hugdahl, 2002b; Grewe et al., 2014). Ocklenburg et al. (2011) reported no significant differences between back and front orientation, whereas Hirnstein et al. (2011) did. This effect is most likely due to back-view stimuli being easier to process, as they do not require a mental rotation, whereas front-view stimuli usually do (Grewe et al., 2014). We also observed an impact of the number of crossings on reaction time, with a significant difference between all types of stimuli (0 vs. 1, 0 vs. 2, and 1 vs. 2 crossings). Ocklenburg et al. (2011) described a similar effect but did not find a significant difference between no crossing and one crossing. This is also related to the difficulty of the task, increasing along with the number of crossings (Ofte and Hugdahl, 2002a), thus increasing the reaction time. A congruency advantage on reaction time was found: Participants were faster to answer “True” than “False.” This effect has never been described in LRD research but exists with the same magnitude (250–300 ms) in some other tasks, such as number categorization with Yes/No answers (Sheridan and Flowers, 2010), numerical reasoning questions (Vamvakoussi et al., 2012) or True/False classification of objects’ properties (Collins and Quillian, 1969), with a faster reaction time for True/Yes over False/No. Thus, this confirmation bias does not appear specific to the task but supports the observation that it is easier to confirm than to deny a proposition, whatever the task.

In agreement with previous reports, we found no main effect of handedness on LRD performance (Ofte, 2002; Gormley et al., 2008; Grewe et al., 2014; Slagman, 2014; McKinley et al., 2015). However, we unraveled an interaction between handedness and the target hand’s laterality. Although right-handers showed no significant difference between any of the conditions, left-handers were better and faster at identifying left target-hands than right target-hands. Interestingly, this better performance of left-handers was not limited to target hands but also applied to the label itself, independently of the target hand. Left-handers were faster when the label “L,” rather than “R,” was presented. This is somewhat in contradiction to Marzoli et al. (2015), who found that left-handers were naturally biased toward the right arm. Our finding is also more consistent with the common-coding hypothesis (Hommel et al., 2001), which proposes that producing actions enhances the perception of related actions or actions that share features (Schütz-Bosbach and Prinz, 2007). It is noteworthy that such facilitating effects were not present in right-handers: right-handers were not faster at identifying a right hand or when a label “R” was presented. The reasons remain unclear, but one can speculate that the fact that the BLRDT bears on an allocentric perspective played a role. As a matter of fact, a comparable advantage of left-handed children over right-handed children in perceiving the left hand from an allocentric perspective has been reported earlier (Etaugh and Brausam, 1978). The authors’ interpretation was that left-handed children are more aware of their handedness and seek left-handedness in others as reassurance of normality, making them more efficient at processing the concept of left. It has been further argued that laterality of others was a more distinctive trait for left-handers than for right-handers (Thompson and Harris, 1978). This observation supports that allocentric perspective would promote the salience of left-handedness in left-handers. Interestingly, in an egocentric perspective, left- and right handers showed mirrored effects. For example, it has been shown that left-handers associated positive abstract notions such as “goodness” or “intelligence” with the left, while right-handers showed the opposite pattern (Casasanto, 2009, 2011).

Women had a significantly higher error rate than men. This is consistent with the reports of several studies (e.g., Ofte and Hugdahl, 2002a; Gormley et al., 2008). It is worth noting that the effect size was small and thus likely to be sensitive to the type of LRD task used, the sampling bias and the factors included in the statistical model. However, we observed an interaction for sex and the number of crossings on reaction times, with women being slower than men as the difficulty increases. This underlines that the sex effect can be rather subtle and could depend on the type and the difficulty of the task performed (Grewe et al., 2014). The picture becomes even more complex in the present study, as a trend was found for not fully lateralized women (MPS-) to be slower than not fully lateralized (MPS-) men, independently of their handedness, as right-handed women MPS- made more errors than right-handed men MPS-. This suggests that sex may interfere with handedness. The hypothesis of reduced brain lateralization in women has been invoked to explain their lower performance (Corballis and Beale, 1970, 1976; Bakan and Putnam, 1974; Hirnstein et al., 2011). Studies have also often investigated the effect of handedness, with the same hypothesis that left-handers would have reduced brain lateralization and would be more prone to Left–Right Confusion (Brandt and Mackavey, 1981). However, several studies that included a large sample of left-handers did not show any difference in hemispheric lateralization related to handedness (Mazoyer et al., 2014; Tzourio-Mazoyer et al., 2015; Mellet et al., 2016; Biduła et al., 2017). In the same way, recent neuroimaging studies (Hirnstein et al., 2011; Hjelmervik et al., 2015) found no reason to believe that sex differences in LRD were related to a more bilateral brain in women. The fact that the effect of sex emerged through complex interaction with handedness in the present study could explain the fact that both effects are inconsistently reported in the literature.

Two domains of cognitive efficiency, namely verbal long-term memory and spatial cognition, were related to shorter reaction times. This fits with the proposition made by Benton that good performance on LRD rely on various cognitive abilities, including language (Benton, 1968). It has been shown that children with verbal learning disabilities have persistent difficulties in LRD (Cermak, 1984), but to our knowledge, this is the first time that a relationship between proficiency in LRD and verbal memory is reported in adults. The implication of a verbal memory component in LRD supports previous results showing that LRD bears on verbal labeling rather than on perceptual encoding (Sholl and Egeth, 1981). Those results emphasized that the association between words and directions is crucial for the emergence of the concept of left and right. Accordingly, it has been shown that students who used specific non-verbal strategies to discriminate left from right (referring to their writing hand, for example) exhibited poorer performance on BLRDT than students who did not rely on any technique (Gormley et al., 2008). An advantage of verbal strategy has also been reported in the practice effect of BLRDT (Grewe et al., 2014). Our results suggest that the association of the words left and right to the corresponding concepts may be more robust and more easily accessible for people with good verbal long-term memory.

Spatial cognition was the other cognitive component related to LRD performance. This finding could appear to contradict some previous studies that reported the absence of relationships with mental rotations or maze tests (Jordan et al., 2006; Ocklenburg et al., 2011). However, it worth noting that the spatial cognition component of the present study represented abilities in processes common to mental rotation, navigational tasks, short-term spatial memory, and spatial reasoning. The association between BLRDT and this component does not extend to each test individually. Accordingly, the reaction times to BLRDT were not associated with the Mental Rotations Test (*p* = 0.45), and the association with the Maze test was only marginal (*p* = 0.08), which is in line with previous reports. Overall, our results suggest that speed performance on BLRDT rely on the fluid aspects of spatial cognition, including executive function, effortful control, and working memory capacity rather than on the specific abilities assessed by each test. This also fits with the proposition that the decline in performance on the BLRDT test observed among elderly participants corresponds to a general cognitive decline rather than being related to specific visuo-spatial operations (Ofte and Hugdahl, 2002a).

It has previously been shown that participants with a bilateral hemispheric involvement in a language production task scored lower than participants with a typical leftward dominance in various cognitive tests, including visuo-spatial assessments (Mellet et al., 2014b). One might speculate that left–right discrimination might be another illustration of this phenomenon. One could indeed relate the hemispheric lateralization for language with performance in BLRDT with participants having the lower score to BLRDT being those with the less pronounced leftward lateralization for language. However, testing this hypothesis would require a large number of participants because this effect, although obvious, was weak (Mellet et al., 2014b).

## Conclusion

Due to this study’s balanced ratio in handedness and sex, we showed an interaction between sex and manual preference, thus providing new insights into the characterization of left–right discrimination variability. We also found a significant advantage of left-handers for the concept of left, whether we tested hand laterality or the label. Finally, an extensive assessment of cognitive abilities allowed us to show that independent of sex, high spatial and verbal long-term memory abilities increased the speed, but not the accuracy, of LRD.

## Author Contributions

EM designed the study. MC performed the experiments. MC and EM analyzed the data and wrote the article.

## Conflict of Interest Statement

The authors declare that the research was conducted in the absence of any commercial or financial relationships that could be construed as a potential conflict of interest.
